# Rapid construction of a dendritic cell vaccine through physical perturbation and apoptotic malignant T cell loading

**DOI:** 10.1186/1476-8518-3-4

**Published:** 2005-07-19

**Authors:** Maria Salskov-Iversen, Carole L Berger, Richard L Edelson

**Affiliations:** 1 Department of Immunology, AArhus University, Aarhus, Denmark; 2 Department of Dermatology, Yale University, School of Medicine, New Haven, CT, USA

## Abstract

We have demonstrated that adherence and release of monocytes from a plastic surface drives their differentiation into immature dendritic cells (DC,) that can mature further during overnight incubation in the presence of apoptotic malignant T cells. Based on these results, we sought to develop a clinically, practical, rapid means for producing DC loaded with malignant cells.

A leukapheresis harvest containing the clonal, leukemic expansion of malignant CD4^+ ^T cells was obtained from the blood of patients with cutaneous T cell lymphoma (CTCL). CTCL cells were purified with a CD3-magnetic bead column where CD3 engagement rendered the malignant T cells apoptotic. The monocyte fraction was simultaneously activated by column passage, re-added to the apoptotic CTCL cells and co-cultured overnight. CTCL cell apoptosis, DC differentiation and apoptotic malignant T cell ingestion were measured by immunostaining.

The results demonstrate that as monocytes passed through the column matrix, they became activated and differentiated into semi-mature DC expressing significantly increased levels of class II, CD83 and CD86 (markers associated with maturing DC) and reduced expression of the monocyte markers CD14 and CD36. Apoptotic malignant T cells were avidly engulfed by the phagocytic transitioning DC. The addition of supportive cytokines further enhanced the number of DC that contained apoptotic malignant T cells.

Functional studies confirmed that column passaged DC increased class II expression as shown by significantly enhanced stimulation in mixed leukocyte culture compared to control monocytes. In addition, DC loaded with apoptotic CTCL cells stimulated an increase in the percentage and absolute number of CD8 T cells compared to co-cultivation with non-loaded DC. After CD8 T cells were stimulated by DC loaded with malignant cells, they mediated increased apoptosis of residual CTCL cells and TNF-α secretion indicating development of enhanced cytolytic function.

We report a simple one-step procedure where maturing DC containing apoptotic malignant T cells can be prepared rapidly for potential use in vaccine immunotherapy. Ready access to both the DC and apoptotic cells provided by this system will allow extension to other malignancies through the addition of a variety of apoptotic tumor cells and maturation stimuli.

## Background

Cutaneous T cell lymphoma (CTCL) is a malignant expansion of mature, clonal CD4 T cells with an affinity for epidermal localization [1]. The tumor cells proliferate in the epidermis around a central Langerhans cell (LC) and previous studies have demonstrated that immature DC play a crucial role in the life cycle of the malignancy [2]. The final stages of CTCL are characterized by systemic spread, immunosuppression and a poor prognosis. Despite the malignancy's dependence on immature DC for proliferative support, DC immunotherapy has been of benefit in this disease [3,4].

Two strategies for the treatment of CTCL, extracorporeal photopheresis (ECP) and transimmunization, have been used to successfully treat this aggressive malignancy [4,5]. The underlying principle of these treatments is extracorporeal establishment and re-infusion of malignant T cell-loaded DC [6]. In both therapies, a leukapheresis product is treated with the drug 8-methoxypsoralen (8-MOP) and passed through a plastic ultraviolet light (UVA) exposure plate. The 8-MOP intercalates in the DNA of nucleated cells and is cross-linked to adjacent pyrimidine bases by UVA light activation. The cross-link formation is a lethal defect and replicating cells are rendered apoptotic. At the same time, monocytes are activated by adherence and release from the plastic exposure plate surface and begin to transition into immature DC [6]. In the ECP treatment, both apoptotic CTCL cells and transitioning DC are re-infused into the patient immediately and association of the DC and apoptotic tumor cells occurs inefficiently *in vivo*.

The transimmunization procedure was devised as a more effective modification of ECP and named to designate the transfer of tumor antigens to competent antigen presenting cells (APC) that could display the full complement of tumor antigens in the context of co-stimulatory and adhesion molecules. In the transimmunization procedure, the apoptotic malignant T cells and the transitioning DC are co-cultured overnight enabling the up-take of the apoptotic cells by the avidly phagocytic immature DC [6]. The activated monocytes produce cytokines that comprise the constituents of monocyte conditioned media thereby, potentiating the maturation of the malignant T cell-loaded DC [3]. The differentiating DC are re-infused the next day into the patient where they can further mature and have the potential to migrate to lymph nodes and induce anti-tumor immunity.

In the current studies, we sought to explore the role of physical perturbation in the monocyte to DC transition by examining whether passage through a separation column that contains a porous matrix is sufficient to induce overnight DC differentiation from monocytes. Studies [7] suggest that trans-migrating monocytes passing through the small spaces of an endothelial cell layer become activated and assume the phenotype of immature DC. This monocyte-to-DC transition can be preserved by phagocytosis of particulate material such as zymosan [7]. We have also previously demonstrated that CD3-binding renders antigen-experienced proliferating CTCL cells apoptotic [2]. We therefore sought to take advantage of the dual observations of the role of physical stimulation in DC maturation and the rapid apoptotic cell death mediated by CD3-binding to develop in one day a clinically practical vaccine. We demonstrate that a simple one-step procedure using CD3-magnetic beads to render the malignant T cells apoptotic and the separation column matrix to simultaneously activate monocytes results in overnight production of apoptotic cell-loaded DC. These immature DC generated in the absence of cytokines could be driven to differentiate further when exogenous cytokines were added. Functional evaluation of the malignant T cell loaded DC, developed by this methodology, demonstrated a significantly enhanced stimulatory capacity in mixed leukocyte culture and the ability to promote CD8 T cell expansion and cytolytic capacity.

Therefore, this approach yields malignant cell loaded DC in a rapid time-frame without extensive cell culture, exogenous factors or cell isolation and manipulation. This method may provide a clinically practical means for the production of immunogenic DC for cancer vaccine therapy.

## Materials and methods

### Patient Population

Therapeutic leukapheresis specimens were obtained from 7 CTCL patients (in accordance with the guidelines of the Yale human investigation committee). All patients had advanced disease with clonal CD4^+ ^T cell populations present in the peripheral circulation as determined by immunophenotyping with antibodies to the clonotypic variable region of family-specific T cell receptor (TCR) or polymerase chain reaction to detect rearrangements of the beta or gamma chain of the TCR. All patients were undergoing treatment with standard ECP.

### Cell Isolation

Mononuclear cells (MNC) were isolated by centrifugation over a ficoll-hypaque gradient followed by two washes in RPMI 1640 (Gibco, Gaithersburg, MD) containing 10% AB serum and 2 mM EDTA. MNC (2 × 10^7^) were incubated with 40 μl Macs α-human CD3 microBeads (Miltenyi Bioteck, Auburn CA) following the manufacturer's directions. The cells were separated by passage through a Macs Separation Column (Miltenyi Bioteck) consisting of a magnetized iron matrix. CD3 positive and negative cells were counted, re-mixed together and incubated overnight. As a control, MNC (2 × 10^7^) were also incubated with 40 μl Macs α-human CD4 microBeads. After treatment, the cells were incubated in 3 ml RPMI 1640 containing 15% AB serum and 15% autologous plasma in one well of a 12 well tissue culture plate (Falcon). In some experiments half of the recombined cells obtained after CD3 column passage were incubated overnight in RPMI containing 10% FCS (Gibco) in the presence of the cytokines GM-CSF 800 U/ml and IL4 1000 U/ml (R & D Systems, Minneapolis, MN). Day 0 baseline cells were immediately removed for immunostaining while Day 1 cells were incubated overnight.

### Immunophenotyping

In order to monitor DC differentiation, the cells were stained by two-color immunofluorescence with a panel of antibodies to monocytes, DC and apoptotic cells. Cells (1 × 10^6^) were incubated with 10–20 μl of fluorocrome conjugated monoclonal antibody for 30 minutes in the dark at 4°C. The antibodies were directly conjugated to fluorescein (FITC) or phycoerythrin (PE) and included: CD14-FITC (monocytes) + CD86-PE (co-stimulatory molecule highly expressed on DC); HLA-DR-FITC (anti-class II MHC molecule) and CD83-PE (DC maturation marker); and their isotype matched controls (Beckman Coulter Immuno-Tech, Hialeah, FL). Cells were washed once and suspended in PBS and read on a XL flow cytometer (Beckman Coulter) within 24 hours.

Combined membrane and cytoplasmic staining was performed following manufacturers instructions (Intraprep kit, Beckman Coulter). Antibody combinations included: membrane CD36-FITC (receptor for apoptotic cells) + cytoplasmic CD83 PE; DR-FITC + cytoplasmic CD83-PE; and isotype controls (Beckman Coulter). To detect apoptotic cells, lymphocytes were stained with: membrane HLA-DR-FITC (class II MHC) + cytoplasmic Apo2.7-PE (apoptotic cells); and isotype controls. Data was analyzed using the CXP software (Beckman Coulter).

### Confocal Microscopy

Cells were double-stained for membrane HLA-DR-FITC + cytoplasmic Apo2.7-PE following the manufacturer's instructions for combined membrane and cytoplasmic staining (see immunophenotyping). In addition, cells were double stained for cytoplasmic LAMP-2 FITC (lysosomal marker, Research Diagnostics) and HLA-DR-PE. Cells were prepared for microscopy following the instructions for Molecular Probes "Slow Fade Light" anti-fade kit (Molecular Probes Inc, Eugene, OR). Specimens were kept in the dark at 4° until microscopy was performed on a Zeiss confocal microscope.

### Mixed leukocyte culture assay

The mixed leukocyte culture assay was performed by isolating control leukocytes from two normal donors. Control T cells were purified with CD4 magnetic beads and the column effluent containing monocytes and B cells was γ-irradiated to prevent differentiation and used as a source of stimulators. Transitioning DC from CTCL patients were obtained one day prior to the normal control cells and cultured overnight without cytokines, γ-irradiated and used as stimulators for the control lymphocytes. The cells were adjusted to 4 × 10^6^/ml and 50 μl of responding cells and 50 μl of stimulating cells co-cultured in round bottom microtiter wells with the addition of 100 μl of RPMI 1640 containing 15% AB serum and 15% autologous plasma for 6 days at 37°C under a 5% CO_2 _atmosphere. The wells were pulsed with 1 μCi/well ^3^[H]-thymidine 16 hours prior to harvest (PhD harvester, Cambridge Tech., Cambridge, MA). The incorporation of the isotope was evaluated in a liquid scintillation counter.

### CD8 T cell purification and expansion

CD8 T cells were purified with CD8-magnetic beads (≥96% purity) and suspended in RPMI 1640/15% autologous serum and IL2 and added to DC that had been column eluted from the same CTCL patient. The cells were co-cultured overnight with 1.1 × 10^6 ^CD8 T cells/well added to CD3-bead rendered apoptotic CTCL cells or viable CTCL cells (4 × 10^6^/well). After overnight culture, the cells were harvested, counted, and immunophenotyped for markers of T cells (CD3, CD4, CD8) and apoptosis (Apo2.7).

### Tumor necrosis-α(TNF-α) ELISA

The production of TNF-α was measured in an ELISA assay (R&D Systems, Minneapolis, MN) essentially as described by the manufacturer.

### Statistical evaluation

The expression of DC markers and the MLC response was evaluated statistically by the student's t test or if the data was not normally distributed the Mann-Whitney Rank Sum Test using the Sigma Stat analysis program.

## Results

### Passage of monocytes through a separation column induces monocyte to DC transition

**Figure 1:**
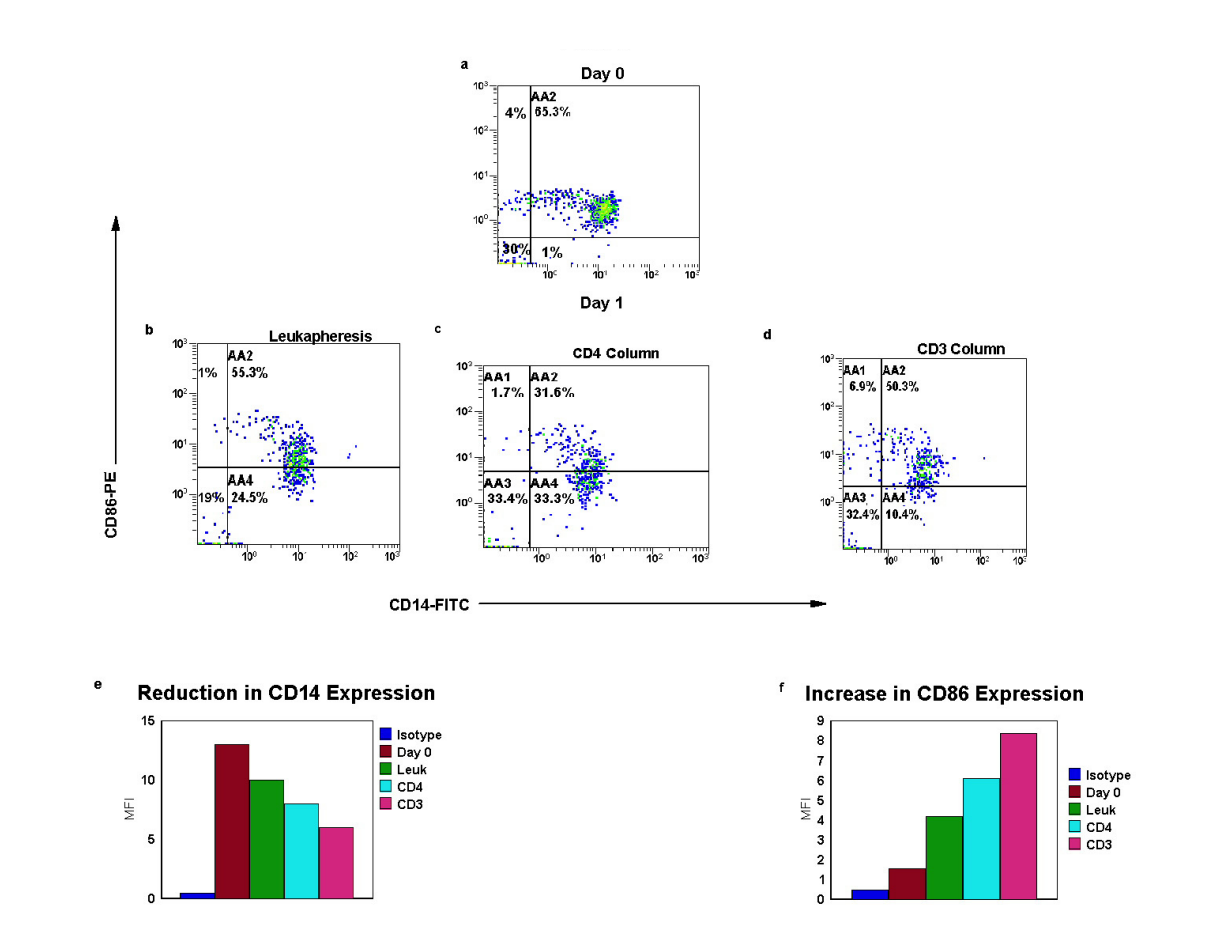
**DC differentiation from monocytes induced by column activation**. CTCL cells and DC were isolated from a leukapheresis by CD4 or CD3-antibody conjugated to magnetic beads. The cells were separated by passage through a column placed in a magnetic field and the purified CTCL cells were re-added to the column activated monocytes and cultured overnight. Binding of fluorochromes was analyzed using flow cytometry and 2-color quadstats were gated on the monocyte population. The results demonstrate membrane CD14-FITC and CD86-PE co-expression on cells obtained **a**: Day 0, primary isolation; and after overnight culture of **b**: leukapheresis cells; **c**: cells obtained by CD4-magnetic bead isolation and re-cultured overnight with column activated monocytes; and **d**: cells obtained from CD3-magnetic bead isolation and re-cultured overnight with column activated monocytes. **e: **Bar graph showing the reduction in mean fluorescent intensity (MFI) of CD14 expression on primary isolation (Day 0) and after overnight incubation of the leukapheresis (leuk) or column passaged and recombined cell populations using CD4 or CD3-magnetic bead isolation (negative control isotype staining is presented in the first bar). **f**: Bar graph showing the increase in MFI of CD86 expression (as described in e).

Monocytes were obtained from a leukapheresis harvest performed therapeutically on CTCL patients and were cultured overnight with and without passage through a magnetic bead separation column. Monocyte differentiation into semi-mature DC was monitored by 2-color immunofluorescence. In a representative experiment, (Fig. 1, gated on the monocyte population as identified by co-expression of CD14 and CD86), the loss of monocyte membrane marker CD14 is revealed by a decrease in the mean fluorescence intensity (MFI) of the CD14 fluorochrome. CD14 expression declined as the degree of manipulation of the cells increased from primary isolation (Fig. 1a) to simple overnight culture of the leukapheresis product (Fig. 1b), compared to the addition to the differentiating DC of CTCL cells that were selected by the CD4 antibody, (Fig 1c) to the maximum reduction in monocyte CD14 expression found when the activated transitioning DC were cultured with CTCL cells rendered apoptotic by CD3 antibody (Fig. 1d). In total, (Fig 1e) the expression of CD14 was reduced by 54%, from a mean fluorescent intensity (MFI) of 13 on primary isolation to 5.89, when the column separated monocytes were co-cultured with the CD3-treated apoptotic CTCL cells. As the differentiating DC lost the monocyte marker, a 3-fold increase in expression of CD86, a co-stimulatory molecule, was found ranging from an MFI of 1.98 on Day 0 to 6.4 after passage through the CD3-magnetic bead column and overnight incubation (Fig. 1f).

### Transitioning DC increase their expression of the maturation marker, CD83

**Figure 2:**
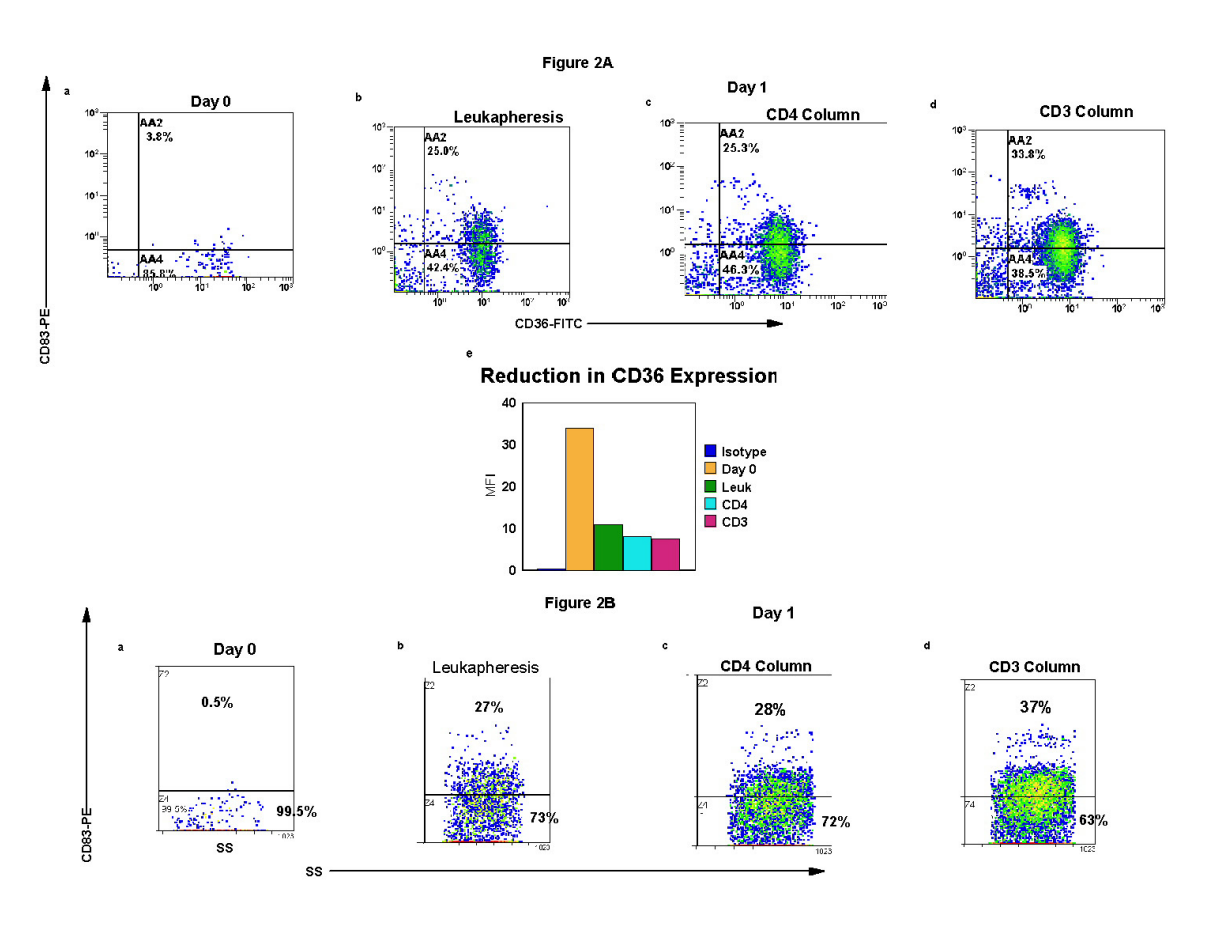
**DC maturation induced after column separation and overnight incubation**. **Fig. 2A**: CTCL cells and monocyte/DC isolated as described in Figure 1 were fixed and permeabilized and stained with CD36-FITC (membrane) and CD83-PE (cytoplasm). The results show 2-color quadstats gated on the monocyte population of cells obtained from **a**: Day 0, primary isolation; after overnight culture of **b**: leukapheresis cells; **c**: CD4-magnetic bead isolation and re-addition to column activated monocytes; **d**: CD3-magnetic bead purification and re-addition to column activated monocytes; **e**: Bar graph of the MFI of membrane CD36 expression on the cell populations. **Fig. 2B**: Demonstration of cytoplasmic CD83 expression in the monocyte/DC population gated by side-scatter (SS) on 100% of the monocyte population. Cell treatment **a–d **as described for Fig 2A.

In Fig. 2A &2B, the differentiation of monocytes into semi-mature DC is demonstrated by an increase in the percentage of cells that exhibit reduced fluorescent intensity of membrane CD36 (receptor for up-take of apoptotic cells, a marker that is lost as DC mature) and increased expression of cytoplasmic CD83 (DC maturation marker). Fig. 2A-a, demonstrates that only 4% of the cells co-express membrane CD36 and cytoplasmic CD83 on primary isolation. When the cells were cultured overnight, the percentage of cells co-expressing CD36/CD83 increased as the level of manipulation rose from 25% in the overnight culture of the leukapheresis (Fig. 2A-b) and in cells separated with a CD4-magnetic bead control antibody and re-added to the column effluent (Fig. 2A-c) to the maximal differentiation of 34% found when apoptotic CD3-treated CTCL cells were re-added to the activated transitioning DC (Fig. 2A-d). In Fig 2A-e, the reduction in CD36 MFI is shown by a decline from a MFI of 34 on primary isolation to 7.7 (77% reduction) in the monocyte/DC population activated by passage through the separation column and recombined for overnight culture in the presence of CTCL cells rendered apoptotic with CD3 antibody.

The increase in cytoplasmic CD83 expression is shown in Fig. 2B. As expected only a small percentage of cells express the DC differentiation marker, CD83 on primary isolation (0.5%, Fig. 2B-a). Overnight incubation of the leukapheresis (Fig 2B-b) increases CD83 expression to an equivalent degree as CD83 expression detected after passage through a CD4-magnetic bead column (Fig. 2B-c). More than one third of the monocytes transitioned into semi-mature DC as shown by the increased expression of cytoplasmic CD83 (Fig. 2B-d) found when CD3-separated apoptotic CTCL cells were added to the column activated monocytes.

### Induction of simultaneous DC differentiation and CTCL cell apoptosis and engulfment

**Figure 3:**
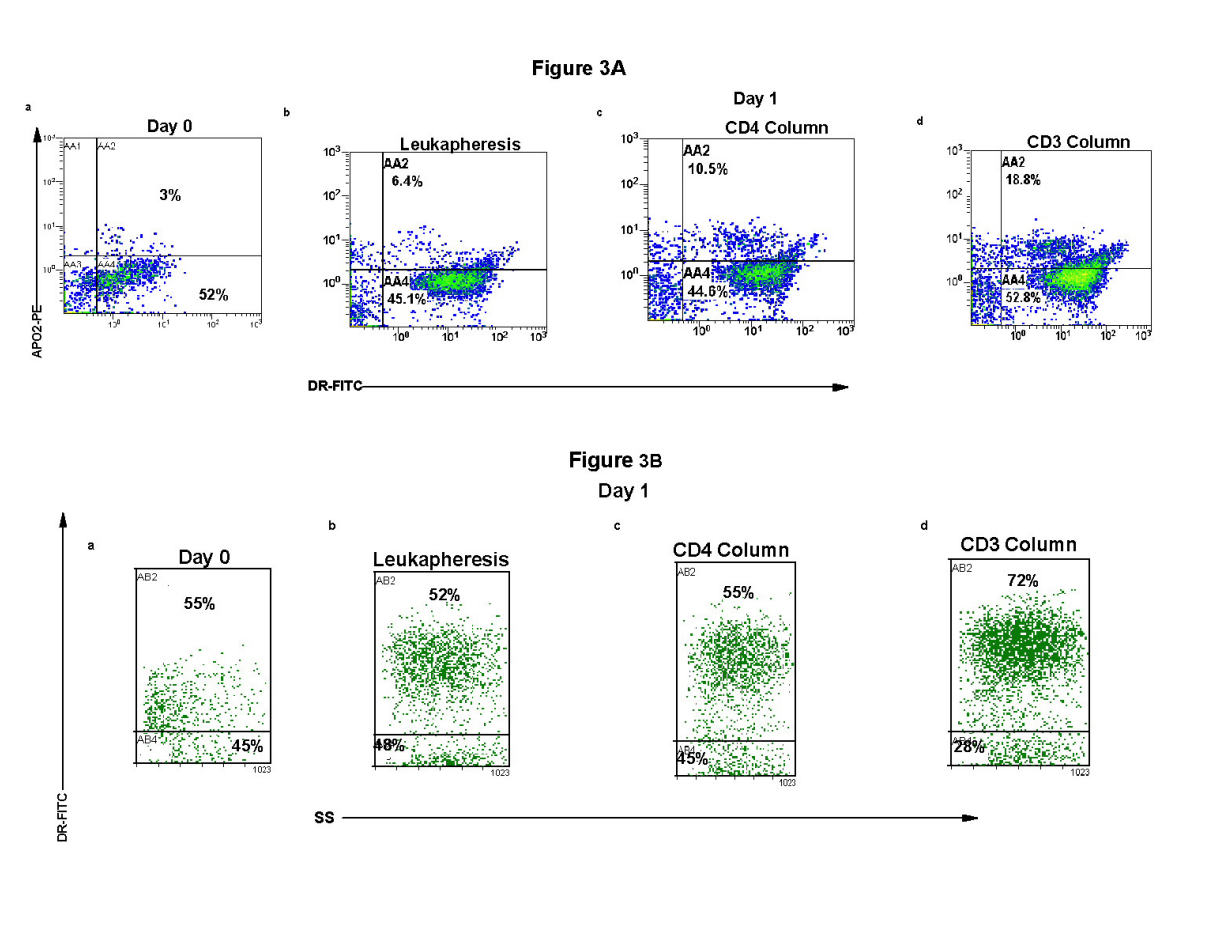
**Increased class II expression on semi-mature DC after ingestion of apoptotic CTCL cells**. **Fig. 3A**: CTCL cells and DC prepared as described in Figure 1 were fixed and permeabilized and stained with DR-FITC (anti-class II MHC antibody, membrane) and APO2-PE (cytoplasm). The results present 2-color quadstats gated on the monocyte population of cells obtained from **a**: Day 0, primary isolation; **b**: leukapheresis cells; **c**: CD4-magnetic bead isolation and re-additon to column activated monocytes; **d**: CD3-magnetic bead purification and re-addition to column activated monocytes. **Fig. 3B**: Membrane DR staining on the monocyte/DC population gated on the total monocyte population by SS. Cell treatment **a–d **as described for Fig 3A.

Further confirmation of enhanced differentiation of monocytes to DC was found when membrane class II expression (HLA-DR) was measured and the up-take of apoptotic CTCL cells was assessed. In figure 3A, the percentage of DR-positive transitioning monocytes containing apoptotic cells was determined by measurement of the cytoplasmic expression of the early apoptotic marker APO2-PE. On primary isolation (Fig. 3A-a), or after overnight incubation of the leukapheresis without further processing (Fig. 3A-b), only a small percentage of the monocyte-DC population contained apoptotic material in the cytoplasm. CD4-treatment and column passage damaged enough cells to increase the number of apoptotic CTCL cells ingested by the activated monocyte-DC population (Fig. 3A-c). As previously reported [2], CD3-binding to CTCL cells rendered the malignant T cells apoptotic and material from the damaged and dying CTCL cells could be detected inside the developing DC population (Fig. 3A-d). While only 19% of the transitioning DC were reactive with DR/APO2-PE, this probably represents only a minimal level of engulfed apoptotic cells since processing and degradation of the apoptotic blebs during overnight incubation could have reduced the detectable expression of APO2-PE positive material.

Differentiation of the DC population was also demonstrated by the increase in expression of membrane class II MHC molecules. Physical manipulation did not increase class II expression from the primary value obtained on initial isolation (Fig. 3B-a), when leukapheresis cells were cultured overnight (Fig. 3B-b). No enhancement of class II expression was noted even when the column activated monocytes were co-cultured overnight with CD4-bead separated CTCL cells (Fig. 3B-c). However, the overnight addition of apoptotic CTCL cells, obtained after CD3-binding, to transitioning DC increased class II expression from 55% (Day 0, Fig. 3B-a) to 72% (Fig. 3B-d).

### Statistical evaluation of the enhanced expression of DC differentiation markers

**Figure 4:**
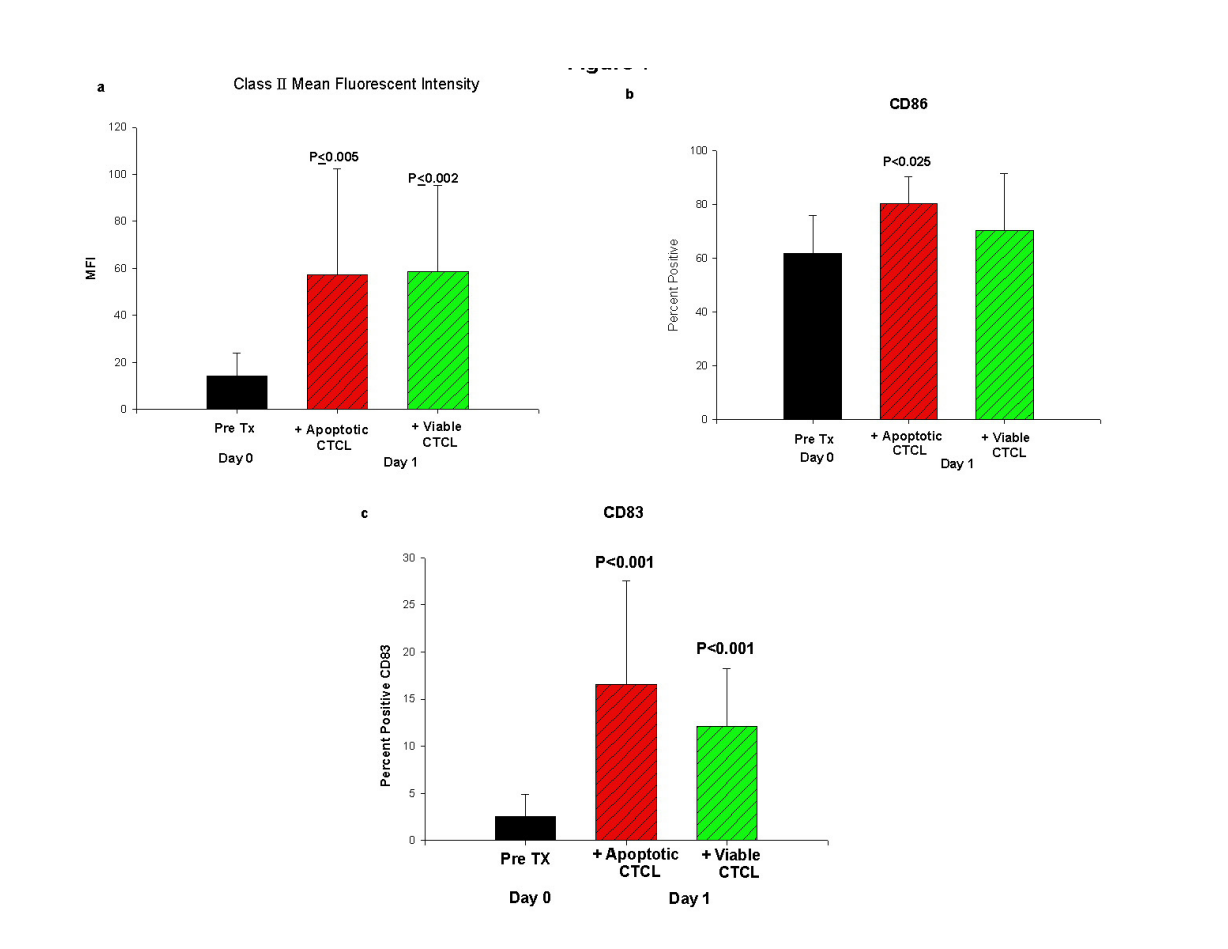
**Statistical analysis of DC differentiation markers**. The expression of markers of DC differentiation were compiled from the overnight culture of DC induced by column passage with and without apoptotic cell loading that had been obtained from 7 CTCL patients, averaged and analyzed for significance in comparison to the values obtained on primary isolation. **a: **Mean fluorescence intensity (MFI) of class II expression on Day 0, primary isolation (Pre Tx; pre-treatment), or Day 1 column activated cells loaded with apoptotic CTCL or co-cultivated in the presence of viable CTCL cells (Mann-Whitney Rank Sum Test). **b: **Percent of monocytes expressing CD86 on primary isolation, or after column activation and overnight culture with and without apoptotic cell ingestion (t test). **c: **Percent of monocytes expressing cytoplasmic CD83 on primary isolation or after column activation and overnight cultivation with and without apoptotic cell up-take (Mann-Whitney Rank Sum Test).

We evaluated the overall increase in markers of DC differentiation from monocytes in leukocytes obtained from seven CTCL patients. While substantial variation in the expression of several antigens precluded analysis, the results showed that overall expression of class II MHC antigen was significantly up-regulated in differentiating DC obtained after column passage with (P ≤ 0.005) and without (P ≤ 0.002) the addition of apoptotic CTCL cells (Fig. 4a). In addition, CD86 (P ≤ 0.025) expression was significantly increased when CTCL cells were co-cultured with column passaged transitioning DC loaded with apoptotic CTCL cells and CD83 (P ≤ 0.001) was enhanced irrespective of the presence of apoptotic CTCL cells (Fig. 4b &4c). These results confirm that the physical perturbation encountered after passage through the small spaces of separation column significantly enhances the entry of monocytes into the DC pathway.

### Demonstration of DC loading with apoptotic cells by confocal microscopy

**Figure 5:**
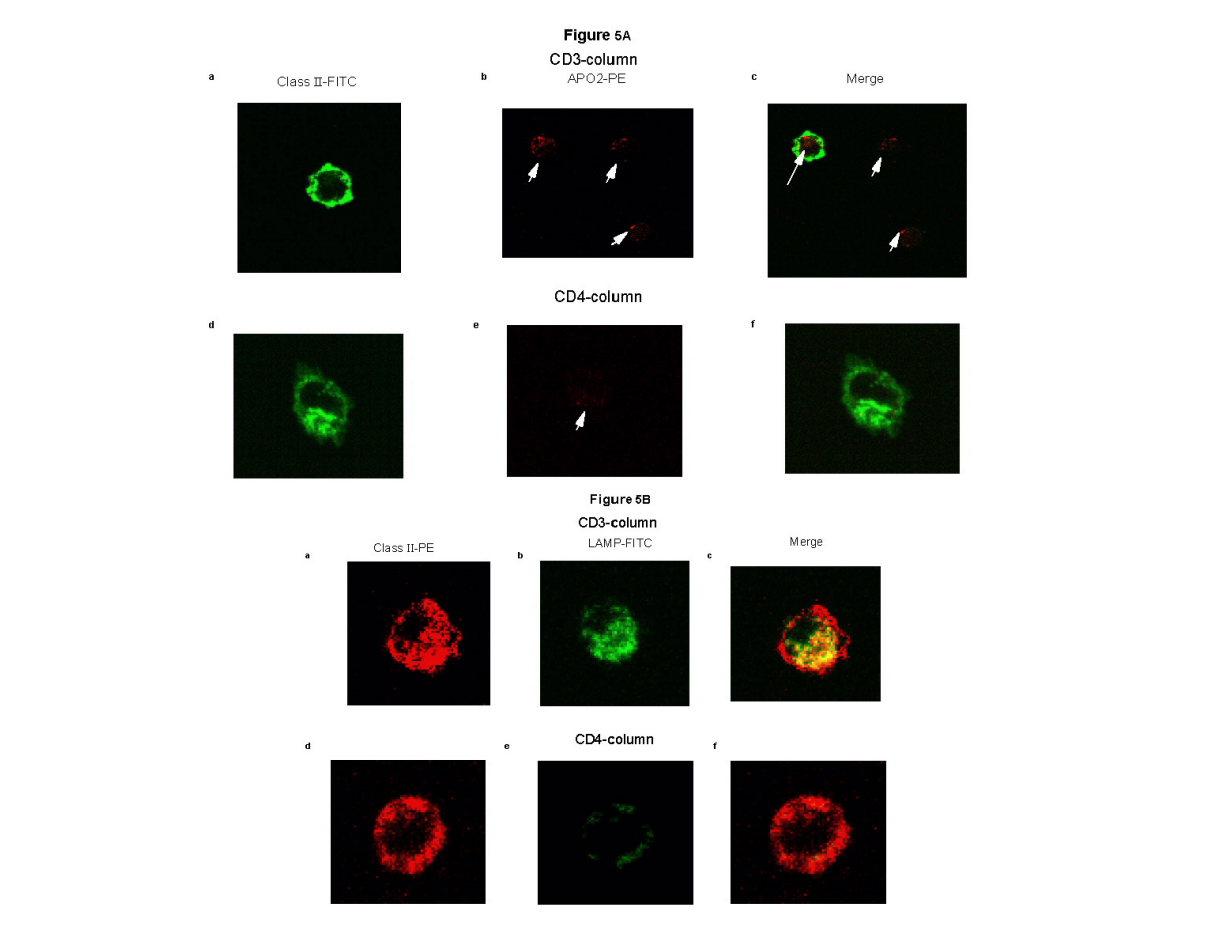
**Confocal microscopic demonstration of apoptotic cell ingestion and class II localization in lysosomal compartments in differentiating DC**. **Fig. 5A: **Cell populations prepared as described in Figure 1 were evaluated by confocal microscopy after fixation and permeabilization and staining. A representative activated monocyte/DC is shown after CD3 column passage and recombination with the apoptotic CTCL cells as detected by **a**: membrane class II-FITC (green); **b**: cytopolasmic APO2-PE (red, white arrows) and **c**: merged image demonstrating internalization of apoptotic material in a class II positive cell. A representative activated monocyte/DC is shown after CD4 column passage and recombination with viable CTCL cells as detected by **d**: membrane class II-FITC (green); e: cytopolasmic APO2-PE (red, white arrow) and f: merged image demonstrating absence of internalization of apoptotic material in a class II positive cell. **Fig. 5B**: Cells prepared as described in Fig. 5A were passed through the CD3 column and stained for **a**: membrane class II-PE (red); **b**: lysosomal membrane marker, LAMP (green); and **c**: merged image showing co-localization of class II molecules in lysosomal compartments. Cells obtained after passage through the CD4 column were stained for **d**: membrane class II-PE (red); **e**: lysosomal membrane marker, LAMP (green); and **f**: merged image showing an absence of co-localization of class II molecules in lysosomal compartments.

In Fig. 5A, CTCL cells were rendered apoptotic with CD3-magnetic bead conjugated antibody (Fig. 5A a–c ) or as a control treated with CD4-magnetic bead conjugated antibody (Fig. 5A d–f), run through the separation column and co-cultured with the simultaneously activated differentiating DC. The activated monocyte/DC population was double-stained for expression of membrane class II (green) and the marker of early apoptotic cells, intracellular APO-2 (red). Representative class II-positive cells (green fluorescence) are seen in Figures 5A-a and 5A-c. In Figure 5A-b, three cells that were rendered apoptotic after CD3-binding, were identified (white arrows) and material from one of these cells is contained in a class II positive cell (merge, Fig. 5A-c). In Figure 5A-e (CD4-treatment), only a small amount of apoptotic material is found and none of this material is associated with the class II positive cell (Fig. 5A-f, merge).

To confirm that class II molecules co-localized in lysosomal compartments in a pattern found in semi-mature DC [8], cells were stained with a lysosomal marker LAMP2 and an antibody to class II MHC molecules (Fig. 5B). In Fig. 5B-a, a cell that has been activated by passage through the separation column and co-cultivated overnight with CTCL cells rendered apoptotic by CD3-magnetic bead binding was stained with an anti-class II antibody (red). In Fig. 4B-b lysosomal compartments were visualized with an antibody that binds to the lysosomal membrane (LAMP2, green). Merging of the 2 fluorochromes (Fig. 5B-c, yellow) demonstrates colocalization of class II MHC molecules in lysosomal compartments. When class II staining was monitored on column activated transitional cells that had been co-incubated with control CTCL cells selected by CD4-magnetic bead separation (Fig. 5B-d, red), strong membrane staining was found. Weak lysosomal staining was localized beneath the plasma membrane (Fig 5B-e, green). When the pictures were merged, class II MHC molecules did not exhibit entry into the lysosomal compartment (Fig. 5B-f). The presence of class II MHC molecules in lysosomes is consistent with differentiation into semi-mature DC [8], and suggests that class II molecules have migrated to lysosomal compartments where they would have the opportunity for loading with peptides derived from processed apoptotic material.

### The addition of supportive cytokines enhances monocyte to DC differentiation

We sought to maximize induction of maturing DC loaded with apoptotic malignant T cells through the addition of exogenous cytokines known to be important for DC differentiation [9]. To study the effect of supportive cytokines on the phenotype of the developing DC, we divided the column separated cells in half and co-incubated them overnight with CD3-bead rendered apoptotic CTCL cells with and without GM-CSF and IL-4.

**Figure 6:**
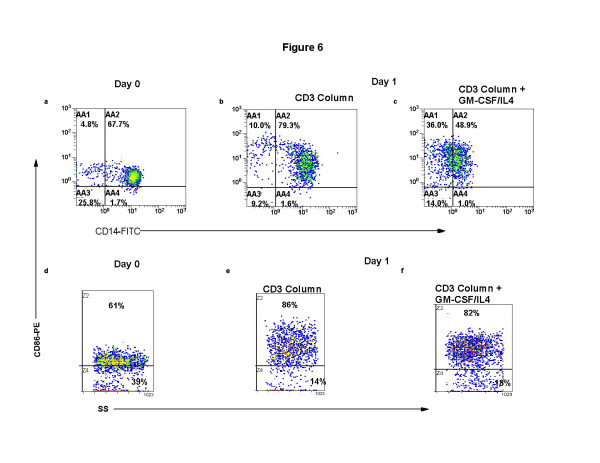
**Exogenous cytokines enhance DC differentiation from monocytes activated by column passage**. Monocyte/DC populations isolated as described in Figure 1 were stained for membrane co-expression of CD14-FITC and CD86-PE. The results present 2-color quadstats gated on the monocyte population of cells obtained from **a**: Day 0, primary isolation; **b**: CD3-magnetic bead purification and re-addition to column activated monocytes; **c**: the same CD3 column purified and activated recombined cell population cultured with the cytokines GM-CSF and IL4. Demonstration of membrane CD86 expression on the monocyte/DC population gated by side-scatter on 100% of the monocyte population. **d**: Day 0, primary isolation; **e**: CD3-magnetic bead purification and re-addition to column activated monocytes; **f**: the same CD3 column purified and activated recombined cell population cultured with cytokines.

The addition of cytokines to the co-cultured apoptotic CTCL cells and column activated transitioning monocytes increased the overall maturation of the DC. In Figure 6, the level of CD14 expression is reduced as shown by an increase in the CD14 negative population (Gate AA1) from 4.8% at baseline (Fig. 6a) to 10% when the transitioning DC were incubated with apoptotic cells without cytokines (Fig. 6b). The addition of cytokines enhanced the loss of CD14 expression resulting in 36% of the cells becoming CD14-negative after overnight culture (Fig. 6c). As the differentiating monocytes lost CD14 expression, a concomitant increase in CD86 expression was noted. CD86 expression rose from a baseline level of 61% (Fig. 6d) to more than 80% CD86-positive transitioning DC after column separation and co-cultivation with CD3-rendered apoptotic cells without cytokines (Fig. 6e) or in the presence of exogenous cytokines (Fig. 6f).

### Cytokines enhance DC maturation

**Figure 7:**
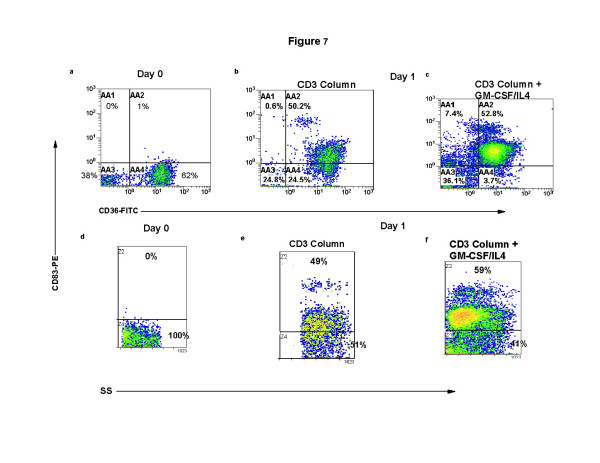
**Exogenous cytokines increase DC maturation induced after column separation and overnight incubation**. Monocyte/DC populations isolated as described in Figure 1 were fixed and permeabilized and stained for expression of membrane CD36-FITC and cytoplasmic CD83-PE. The results present 2-color quadstats gated on the monocyte population of cells obtained from **a**: Day 0, primary isolation; **b**: CD3-magnetic bead purification and re-addition to column activated monocytes; **c**: the same CD3 column purified and activated recombined cell population cultured with the cytokines GM-CSF and IL4. Demonstration of cytoplasmic CD83 expression in the monocyte/DC population gated by side-scatter on 100% of the monocyte poulation. **d**: Day 0, primary isolation; **e**: CD3-magnetic bead purification and re-addition to column activated monocytes; **f**: the same CD3 column purified and activated recombined cell population cultured with cytokines.

**Figure 8:**
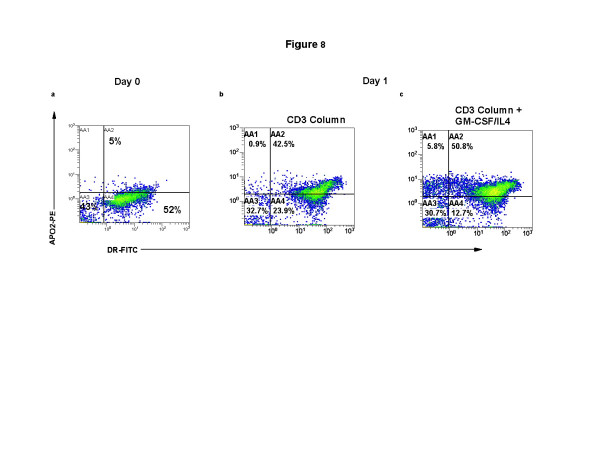
**Exogenous cytokines enhance ingestion of apoptotic material in differentiating DC**. CTCL cells and monocyte/DC populations isolated as described in Figure 1. The cells were fixed and permeabilized and stained for expression of membrane DR-FITC and cytoplasmic APO2-PE. The results present 2-color quadstats gated on the monocyte population of cells obtained from **a**: Day 0, primary isolation; **b**: CD3-magnetic bead purification and re-addition to column activated monocytes; **c**: the same CD3 column purified and activated recombined cell population cultured with the cytokines GM-CSF and IL4.

The percentage of semi-mature DC differentiated after overnight co-culture that co-expressed membrane CD36 and intracytoplasmic CD83 was enhanced by the addition of cytokines. In Fig. 7a, on primary isolation the monocytes expressed intermediate levels of CD36 and did not contain cytoplamic CD83 (Fig. 7a). Co-expression of CD36/CD83 (Fig. 7b) rose to 50%, after overnight culture in the absence of cytokines, on differentiating DC that had passed through the separation column and were recombined with CD3 rendered apoptotic CTCL cells. This increased expression of a receptor for apoptotic cells may have been driven by the presence of very high levels of apoptotic material in the co-cultures (Fig. 8). Further maturation was observed in the presence of cytokines (Fig. 7c) leading to 53% CD36 expression on the transitioning DC and the identification of 7% CD36-negative cells that contained CD83 in the cytoplasm. The percentage of differentiating DC that expressed cytoplasmic CD83 rose from 0% at baseline (Fig. 7d) to 49% after column separation and co-incubation with CTCL cells rendered apoptotic by CD3-magnetic bead binding (Fig. 7e) to 59% when cytokines were added to the cultured cells (Fig. 7f).

### Class II expression and up-take of apoptotic material is enhanced in the presence of cytokines

The baseline expression of class II MHC molecules on the cell membrane of monocytes on primary isolation is shown in Fig. 8a. Freshly isolated monocytes express a reduced intensity of class II expression and contain a small percentage of cytoplasmic apoptotic material. After column separation and co-incubation with CD3-magnetic bead treated apoptotic cells, membrane class II expression is enhanced (Fig. 8b) and large amounts of apoptotic material can be detected in the cytoplasm of the transitioning DC. Exogenous cytokines further increase the percentage of class II-positive cells that contain apoptotic material (Fig. 8c). Therefore, the addition of exogenous cytokines enhances both the differentiation of immature DC and the ingestion of apoptotic material improving the overnight yield of maturing apoptotic T cell loaded DC.

### Functional analysis of the differentiating DC obtained after column passage

**Figure 9:**
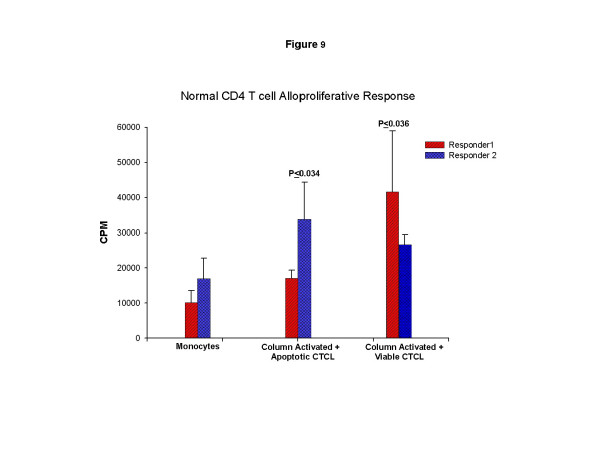
**Mixed leukocyte culture response of normal T cells to monocytes or column activated loaded and non-loaded DC**. Normal CD4 T cells were magnetic bead column purified from the peripheral blood of 2 controls and stimulated with column effluent monocytes that had been γ-irradiated to prevent differentiation, or γ-irradiated CTCL cell monocytes that had been column purified and either loaded or not with apoptotic malignant T cells and cultured overnight one day prior to the normal T cell isolation. The results are presented as delta CPM (less background proliferation obtained by autostimulation) of ^3 ^[H]-thymidine incorporation measured at day 6. Significance was evaluated with a student's t test.

Transitioning DC obtained after column passage were evaluated for their stimulatory capacity in MLC (Fig. 9). The results demonstrate that DC induced by column passage of leukocytes from two normal controls were significantly better stimulators (P ≤ 0.034 & P ≤ 0.036) in MLC than autologous monocytes irrespective of apoptotic cell loading. These results confirm that column activation of monocytes and overnight culture enhances the membrane expression of class II MHC molecules recognized by alloresponsive CD4 T cells. Therefore, DC harvested after physical activation and overnight culture could expand CD4 T cells and potentially provide the help required for licensing of anti-tumor CD8 T cell responses [10].

**Figure 10:**
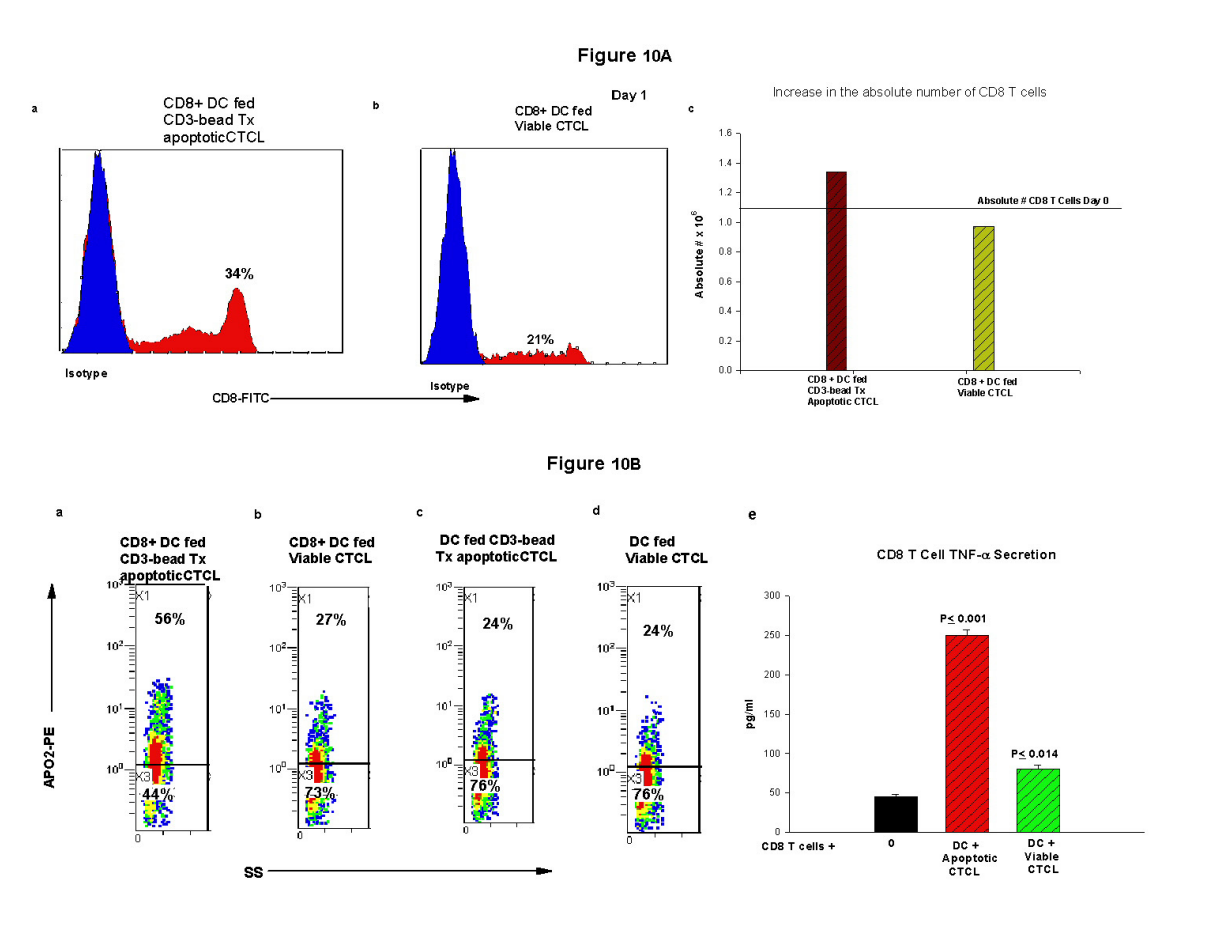
**CD8 T cell response to column-activated malignant T cell loaded DC**. **Fig. 10A: **CD8 T cells were magnetic-bead enriched (≥96% CD8^+ ^T cells) from the leukapheresis of a CTCL patient and added to column-activated DC with and without apoptotic malignant T cell loading. The percentage of CD8 T cells was identified by immunophenotyping and flow cytometry and the results presented as 1-color histograms. **a**: Percentage of CD8 T cells found after overnight culture with column-activated DC pulsed with CD3-magnetic bead rendered apoptotic CTCL cells. **b**: Percentage of CD8 T cells identified after overnight culture with DC co-cultivated with viable CTCL cells. **c**: Absolute number of CD8 T cells after overnight cultivation with DC loaded with apopototic malignant T cells or DC co-incubated with viable CD4-bead isolated CTCL cells. Horizontal line indicates the initial number of CD8 T cells added to the co-cultures on Day 0. **Fig. 10B**: The percentage of apoptotic cells was determined in the co-cultures by staining for APO2.7 and flow cytometry. The quadstats are gated on the lymphocyte population by side scatter (SS) and represent 100% of the lymphocyte population. Percent apoptotic cells found in co-cultures of **a: **CD8 T cells and DC loaded with apoptotic malignant T cells; **b: **CD8 T cells, DC and viable CTCL cells; **c: **DC loaded with apoptotic CTCL in the absence of CD8 T cells; **d: **DC cultured with viable CTCL without CD8 T cells. **e: **Culture supernatants were obtained from CD8 T cells cultured overnight alone, or in the presence of DC loaded with apoptotic CTCL cells or DC cultured with viable CTCL cells and the secretion of TNF-α determined in an ELISA assay. The results are presented as pg/ml and significance determined with a student's t test.

We have begun to investigate the capacity of the DC harvested after column perturbation and apoptotic malignant T cell loading to induce and expand an anti-tumor CD8 T cell response. In these initial studies (Fig. 10A), we have found that the percentage of CD8 T cells (purified CD8 T cells ≥96% positive, obtained from the leukapheresis of a patient responsive to ECP) increased by 38% in the presence of DC fed apoptotic CTCL cells (Fig. 10A-a) compared to the percent of CD8 T cells found after overnight incubation with DC exposed to viable CTCL cells (Fig. 10A-b). In addition, the absolute number of CD8 T cells recovered from the overnight culture of differentiating column passaged DC loaded with apoptotic malignant T cells increased by 22% when compared to the initial number of CD8 T cells while the absolute number of CD8 T cells present in cultures of DC and viable CD4 T cells fell by 12% (Fig. 10A-c). Therefore, exposure of CD8 T cells to malignant T cell loaded DC increases both the percentage and absolute number of potential anti-tumor responsive T cells.

The level of apoptosis found when CD8 T cells were cultured overnight with column activated-DC loaded with CTCL cells doubled (56%, Fig. 10B-a) in comparison to the level of apoptosis present when CD8 T cells were added to non-loaded DC that had been cultivated with viable CTCL cells (27%, Fig. 10B-b). The baseline level of apoptosis was 24% when malignant T cell loaded DC (Fig. 10B-c), or non-loaded DC (Fig. 10B-d) were cultured in the absence of CD8 T cells. These results indicate that residual CTCL cells may be lysed in the presence of CD8 T cells stimulated with DC that have ingested apoptotic malignant T cells.

Finally, further support for the contention that functional CD8 T cells were expanded by overnight exposure to column-activated DC loaded with malignant T cells was obtained by evaluation of the levels of TNF-α found in the culture supernatants. In Figure 10B-e, supernatants from CD8 T cells cultured overnight alone contained minimal levels of TNF-α. CD8 T cells stimulated with column differentiated DC loaded with CD3-bead rendered apoptotic malignant T cells or not loaded both significantly (P ≤ 0.001 & P ≤ 0.014) stimulated release of TNF-α. However, DC that had engulfed apoptotic cells caused the release of three fold more TNF-α than non-loaded DC, indicating that CD8 T cell activation had occurred and the release of a molecule that promotes tumor cytolysis was present.

## Discussion

Development of effective DC based cancer vaccine technology has been limited by the extensive manipulation and extended period of *in vitro *culture required for generation of mature DC loaded with the appropriate tumor antigens. We have circumvented some of these limitations through modification of a successful technology that permits both DC differentiation from peripheral monocytes and simultaneous loading of DC with apoptotic malignant T cells containing the full complement of potential tumor antigens [6]. DC are the most potent APC displaying when mature high levels of co-stimulatory, adhesion and MHC molecules which can present peptides derived from apoptotic cells to the immune system [9]. Therefore, the development of a simple rapid means of generating malignant cell-loaded DC could advance the immunotherapy of CTCL and perhaps other malignancies.

Immunotherapy has played a major role in the treatment of CTCL since the introduction of ECP by Edelson and colleagues in 1987 [5]. The mechanism underlying the success of ECP treatment was defined by the demonstration that the simultaneous introduction of apoptotic malignant T cells and the differentiation of monocytes into DC resulted in patients receiving CTCL cell-loaded DC that have the capacity to present antigen, derived from the CTCL cells, to cytotoxic lymphocytes and initiate an immune response towards the malignant CD4 T cells. Previous studies had demonstrated that despite the clonal expansion of CD4^+ ^malignant T cells in the peripheral blood of CTCL patients, circulating populations of CD8 T cells that retained the capacity to lyse autologous malignant T cells [11] could be identified. One antigen that served as an immunogen recognized by cytotoxic T cells in CTCL was determined to be peptides derived from the beta chain of the TCR that was clonotypically displayed on the malignant T cells [12,13]. Therefore, the potential for development of an anti-malignant T cell immune response exists in CTCL patients and immunotherapeutic approaches designed to expand anti-tumor CD8 T cells could be effective in this disease.

We sought to exploit our understanding of the mechanism of ECP to develop more efficient, rapid, clinically practical means for producing malignant T cell-loaded DC. In the current study, we demonstrate that DC loaded with apoptotic cells can be produced in one day without extensive manipulation or the use of exogenous cytokines. The use of CD3-antibody to render CTCL cells apoptotic and passage of the treated MNC through the small pores of the iron matrix of a separation column followed by overnight co-incubation resulted in the generation of DC containing material derived from apoptotic CTCL cells. DC differentiation was demonstrated by both the reduction in monocyte markers and the significant increase in class II MHC molecules and co-stimulatory molecules, as well as the increase in CD83, a marker of maturing DC. The internalization of apoptotic blebs was confirmed by localization of the apoptotic material in the cytoplasm, indicating that processing of the apoptotic CTCL-derived material could make peptides available for MHC loading and transport to the cell membrane [14]. The ability to increase the number of maturing CTCL cell-loaded DC by the addition of exogenous cytokines demonstrates that this technique can produce cell populations that can be manipulated to maximize the production of DC that contain apoptotic material thereby providing access to a spectrum of CTCL cell-derived epitopes, without the requirement for identification or isolation of individual peptides that may be relevant for induction of an anti-CTCL cell immune response.

Furthermore, we show that DC produced in this fashion are effective stimulators of alloproliferation in MLC confirming the significant up-regulation of class II MHC molecules. The malignant T cell loaded DC stimulated CD8 T cell expansion and an increase in apoptotic cell death and the significantly enhanced release of TNF-α. These results indicate that CD8 T cells that have been stimulated by malignant T cell loaded DC, produced by this methodology, may develop the ability to mediate tumor cell cytolysis.

The current studies support our previous results demonstrating that monocyte differentiation into DC could be driven by increasing levels of physical perturbation [6]. We confirm that leukapheresis alone generates modest monocyte activation and conversion into immature DC that can be enhanced by further manipulation and the addition of apoptotic cells. We also demonstrate that CD3-binding is a potent means of rendering CTCL cells apoptotic [2] even when the CTCL cells are not cultured but directly isolated from the patients. The current study combines and extends these two previous observations into a format for simple, rapid, clinically practical DC vaccine generation.

Current approaches to DC vaccine technology include peptide pulsing [15], one week or longer of culture with cytokines [16], cell fusion with tumor cell partners [17], and the use of a variety of vectors designed to introduce tumor antigens into the DC [18]. These methods are generally cumbersome, require extensive *in vitro *manipulation, and are limited to a small set of known tumor epitopes that may be lost from the patient's tumor, due to immuoselective pressures. Clinical results with these techniques have been variable and seldom provide long-term responses [19]. In contradistinction, treatment of CTCL patients with ECP has demonstrated an excellent safety profile and in multiple studies in the literature an overall response rate for all stages of the disease of 55.7% and a complete response rate of 17.6% [20]. Pilot studies using transimmunization to enhance the interaction of apoptotic tumor cells and differentiating DC through simple overnight incubation has demonstrated encouraging results in some patients [4], that suggest that the therapy retains the safety profile of ECP but may be more potent and effective in a shorter time course.

The technology proposed in this study is likely to be as safe as transimmunization and ECP since it retains the same features of limited cellular manipulation and culture. The replacement of 8-MOP with CD3 antibody should not lead to significant apoptotic cell death and potential tumor lysis syndrome since CD3-binding renders only 30% of the CTCL cell population apoptotic [2]. Since the CD3 antibody is conjugated to the magnetic beads any free antibody could be removed by a second passage through the magnet prior to re-infusion, thereby, limiting the induction of anti-CD3 antibodies. However, presentation of portions of the CD3 antibody after DC ingestion may provoke an immune response that could prevent further therapy. These potential safety issues will require careful monitoring in future clinical trials.

The current results demonstrate that further development of this technology through passage over a column that permits the one-step apoptotic cell death of CTCL cells, sparing of normal cells and activation of monocytes into the DC pathway may further improve the immunogenicity of the reinfusate. Since only proliferating tumor cells are rendered apoptotic by the CD3 antibody, normal resting lymphocytes will not be impacted which is in contrast to the use of 8-MOP/UVA that targets the DNA of all nucleated cells. This preservation of normal T cells may serve to improve the induction of anti-CTCL immune responses to the re-infused apoptotic cell-loaded DC by preventing damage to by-stander normal cells and precluding their uptake that could lead to tolerance induction [21].

Using a peristaltic pump it should be possible to rapidly flow a leukapheresis product through a magnetic separation column. Due to the concentration of MNC obtained with the leukapheresis procedure, high yields of monocytes approaching 10^8 ^cells could be obtained and activated by this procedure [6]. Since CTCL patients have large populations of circulating malignant T cells (approaching >90% of the lymphocyte population), CD3-treatment would provide substantial apoptotic tumor cells for DC loading. Because both activated monocytes and apoptotic malignant T cells are obtained individually and can be re-added after treatment, the optimal conditions for apoptotic T cell and DC co-cultivation can be determined empirically. This access to both cell populations would permit the opportunity for loading DC with other tumor antigens, including solid tumors rendered apoptotic by irradiation or other methods.

Other studies have determined that physical separation of DC clusters by simple pipetting [22] or cell transfer [8,23] is among the most potent means of inducing DC maturation. Furthermore, even semi-mature DC are effective at cross-priming peptide [22] derived from exogenous material into the class I pathway for presentation to CD8 T cells. Our simple approach to rapid DC vaccine construction takes advantage of both physical stimulation and production of apoptotic material providing access to a broad spectrum of CTCL antigens for cross-priming into the class I pathway.

Further studies to determine the functional ability of the CTCL cell-loaded DC produced by this methodology will be required to confirm the immunogenicity of the proposed vaccine components. We have already demonstrated that DC loaded with apoptotic malignant T cells are potent immunostimulators in mixed leukocyte culture [6], can provoke positive clinical results in treated patients [4] and that responsive patients treated by standard ECP develop increased levels of circulating CD8 T cells [24]. The current results indicate that the development of DC loaded with apoptotic cells for use in immunotherapy can be performed in a rapid, simple, clinically practical manner that provides ready access to the major cell types so that additional strategies to optimize the vaccine components can be implemented and monitored prior to re-infusion.

## Competing interests

Drs Berger, Edelson and Yale University hold patents pertaining to the transimmunization procedure.

## Authors' contributions

Maria Salskov-Iverson has performed the majority of the experiments presented in this manuscript and prepared the primary draft of the paper. Drs Berger and Edelson have defined the preliminary observations upon which this manuscript is based and provided intellectual guidance and supervision for the reported work and manuscript.
